# Fabrication of Doxorubicin-Loaded Lipid-Based Nanocarriers by Microfluidic Rapid Mixing

**DOI:** 10.3390/biomedicines10061259

**Published:** 2022-05-27

**Authors:** Chia-Ying Lee, Tsuimin Tsai, Po-Chun Peng, Chin-Tin Chen

**Affiliations:** 1 Department of Biochemical Science and Technology, National Taiwan University, No. 1, Sec. 4, Roosevelt Road, Taipei 10617, Taiwan; d07b22006@ntu.edu.tw; 2 FormuRx Pharmaceuticals Co., Ltd., Taipei 10617, Taiwan; tmtsai00@gmail.com (T.T.); swaigod@hotmail.com (P.-C.P.)

**Keywords:** lipid-based nanocarriers, liposome, doxorubicin, microfluidics

## Abstract

Doxorubicin (Dox) is a widely known chemotherapeutic drug that has been encapsulated into liposomes for clinical use, such as Doxil^®^ and Myocet^®^. Both of these are prepared via remote loading methods, which require multistep procedures. Additionally, their antitumor efficacy is hindered due to the poor drug release from PEGylated liposomes in the tumor microenvironment. In this study, we aimed to develop doxorubicin-loaded lipid-based nanocarriers (LNC-Dox) based on electrostatic interaction using microfluidic technology. The resulting LNC-Dox showed high loading capacity, with a drug-to-lipid ratio (D/L ratio) greater than 0.2, and high efficacy of drug release in an acidic environment. Different lipid compositions were selected based on critical packing parameters and further studied to outline their effects on the physicochemical characteristics of LNC-Dox. Design of experiments was implemented for formulation optimization. The optimized LNC-Dox showed preferred release in acidic environments and better therapeutic efficacy compared to PEGylated liposomal Dox in vivo. Thus, this study provides a feasible approach to efficiently encapsulate doxorubicin into lipid-based nanocarriers fabricated by microfluidic rapid mixing.

## 1. Introduction

Lipid-based nanocarriers (LNCs) can enhance the bioavailability of active pharmaceutical ingredients and alter their biodistribution via encapsulation of drugs inside the nanocarriers [1,2,3]. Drugs loaded inside LNCs can be delivered more efficiently to the target tissue while reducing the side effects. Doxorubicin (Dox) is a potent antineoplastic agent that is active against numerous human cancers. Many methods have been studied for the encapsulation of doxorubicin inside LNCs to alter its biodistribution and biological activity. Encapsulation based on electrostatic interaction has been studied for loading doxorubicin inside nanocarriers, indicating low drug-loading capacity [4,5]. To increase the drug-loading capacity, doxorubicin can be encapsulated into liposomes by using a remote loading approach, which is driven by a transmembrane pH or ion gradient across the liposomes. Doxil^®^, a well-known liposomal formulation used in clinical practice, was prepared via the encapsulation of doxorubicin in the aqueous core as Dox-sulfate crystals using an ammonium sulfate gradient [6]. The high drug-to-lipid ratio (D/L ratio) of Doxil^®^ relies on the counterions used during preparation. However, several studies have shown that the antitumor activity of Doxil^®^ might be hindered by the poor release of the drug from liposomes [7,8]. 

The production of liposomal drugs is usually a multistep process that is easier to achieve within small-scale laboratories. However, it becomes more complicated and challenging to produce reproducible batches of liposomes with identical properties and in sufficient quantities for preclinical and clinical development [9,10]. The ethanol injection method is the preferred method for large-scale production of liposome, and requires the extrusion process for further size control. Several steps are involved in this highly complex manufacturing process; therefore, it is difficult to ensure batch-to-batch reproducibility [11]. Hence, scaling up becomes a challenge to the robust development of liposomal drugs. Microfluidics has shown its great potential for robust and continuous production of lipid-based nanocarriers [12]. Microfluidic rapid mixing is a bottom-up fabrication technique that can produce LNCs of limited size, starting from the molecular level, through the condensation of phospholipids and other excipients from solution [13,14]. The traditional top-down approach often requires mechanical methods, such as sonication and homogenization, to crush the bulk into several parts to form nanoparticles [15]. Microfluidic technology, on the other hand, can generate the laminar flow in the microchannel, which shows high controllability of the mixing processes, resulting in reduced batch-to-batch variability [16,17]. Several operating variables—including the process parameters and material attributes—can be included in the production of LNCs by microfluidic rapid mixing [18]. To facilitate the development of LNCs, a systematic approach is required to outline the relationships and effects between production parameters and the characteristics of LNCs. 

Response surface methodology (RSM) is a mathematical and statistical approach that generates a polynomial equation to fit the experimental data in order to study the multifactorial interaction between the variables [19,20]. RSM has been applied for formulation optimization to understand the effects of formulation variables (independent factors) and the related actions between factors on the responses (dependent factors) [20]. Box–Behnken design (BBD) is thought to be one of the experimental designs for RSM that is helpful for statistical optimization [21]. RSM combined with BBD offers an effective way to achieve the best system performance for the formulation development.

In this study, we aimed to develop a Dox-loaded LNC delivery system (LNC-Dox) based on electrostatic interaction via microfluidic technology. RSM combined with the BBD design scheme was implemented to study the critical attributes of lipid compositions and microfluidic parameters for the optimization of LNC-Dox. The in vivo antitumor effect was tested to further examine the therapeutic efficacy of LNC-Dox in a C26 syngeneic BALB/c tumor model.

## 2. Materials and Methods

### 2.1. Materials

Egg phosphatidylglycerol (EPG), 1,2-dioleoyl-sn-glycero-3-phospho-(1’-rac-glycerol) (DOPG), 1,2-dioleoyl-sn-glycero-3-phosphate (DOPA), L-α-phosphatidylcholine (HSPC), 1,2-dioleoyl-sn-glycero-3-phosphoethanolamine (DSPE), 1,2-distearoyl-sn-glycero-3-phosphoethanolamine (DOPE), and 1,2-distearoyl-sn-glycero-3-phosphoethanolamine-N-[amino(polyethylene glycol)-2000] (DSPE-PEG2000) were purchased from Avanti Polar Lipids, Inc. (Alabaster, AL, USA). Cholesterol was purchased from Merck (Darmstadt, Germany). Doxorubicin HCl and Doxil^®^ were obtained from Taiwan Liposome Company (Taipei, Taiwan). Ethanol was purchased from Taiwan Sugar Corp. (Tainan, Taiwan). Modified polyethersulfene (mPES) hollow-fiber columns with a molecular weight of 100 kDa were purchased from Spectrum Inc. (Breda, The Netherlands). Float-A-Lyzer^®^ dialysis tubes with a 100 kDa molecular weight cutoff (MWCO) were purchased from Spectrum Laboratories (Rancho Dominguez, CA, USA).

### 2.2. LNC-Dox Production

The NanoAssemblr^®^ Benchtop from Precision NanoSystems Inc. (Vancouver, BC, Canada) was used in this study for the preparation of LNC-Dox. To summarize, LNC-Dox was prepared by mixing neutral lipids (HSPC, DSPE, or DOPE), anionic phospholipids (EPG, DOPG, and DOPA), cholesterol, and DSPE-PEG2000 in ethanol with an aqueous solution containing 1 mg/mL solubilized doxorubicin HCl, using a microfluidic system. Production speeds from 1 mL/min up to 12 mL/min were tested. The flow rate ratio (FRR) in microfluidic mixing was controlled in a range from 1.5:1 to 4:1 (aqueous: organic phase). A tangential flow filtration (TFF) system was used immediately for further dialysis and concentration after the preparation of LNC-Dox by microfluidic rapid mixing. The system was run at 28 mL/min with an mPES filter. The final LNC-Dox was stored at 4 °C for further analysis.

### 2.3. Lipo-Dox Production

Doxorubicin was loaded inside liposomes (Lipo-Dox) using an ammonium sulfate gradient. Briefly, the empty liposomes were prepared by mixing 250 mM ammonium sulfate solution and the lipids of HSPC, cholesterol, and DSPE-PEG2000 in ethanol, using a microfluidic system. The total flow rate of the microfluidic system was set at 12 mL/min, and the flow rate ratio of the aqueous and organic phases was 4:1. After the rapid mixing, the liposomes were passed through a size-exclusion chromatograph using a Sephadex G-50 column to replace the buffer with 0.9% NaCl. Then, 1 mg/mL doxorubicin was added to the empty liposomes and incubated at 65 °C for 30 min. The untrapped free-form doxorubicin was removed via size-exclusion chromatography using a Sephadex G-50 column. The final Lipo-Dox was stored at 4 °C for further study.

### 2.4. Particle Size Measurement

Dynamic light scattering (DLS) was used to measure the hydrodynamic mean diameter and the polydispersity index (PDI) of LNC-Dox. The scattered light intensity of the solution at 173° was determined using an SZ-100 nanoparticle analyzer (HORIBA, Kyoto, Japan). Samples were prepared by diluting with deionized water at room temperature. The results were expressed as the mean ± S.D of triplicate samples.

### 2.5. Quantification of Doxorubicin

The UV–Visible method was used for the quantification of doxorubicin encapsulated in lipid nanocarriers. Briefly, LNC-Dox was broken down by adding EtOH. Analysis of the amount of doxorubicin in each sample was carried out at 440 nm in a single wavelength mode with a DU 800 UV–Vis spectrophotometer (Beckman Coulter, Brea, CA, USA). Standard calibration curves were set up by serial dilution of known amounts of doxorubicin. To determine the cellular amounts of doxorubicin, the fluorescence intensity of doxorubicin was monitored at an excitation/emission wavelength of 470/586 nm with a florescent spectrofluorometer (FluoroMax^®^-4, Horiba Jobin Yvon, Edison, NJ, USA).

### 2.6. Drug-to-Lipid Ratio and Entrapment Efficiency Analysis

The loading capacity of LNC-Dox was expressed as the drug-to-lipid ratio (D/L ratio), which was calculated as the amount of drug encapsulated inside the LNCs relative to the total amount of lipid. The Bartlett assay was adopted for the quantification of the phospholipid content of LNC-Dox [22]. The amounts of encapsulated drugs and phospholipids were quantified after LNC-Dox was dialyzed by the TFF system. The D/L ratio was calculated as follows:

(1) $$D/L\;\mathrm{ratio}=\frac{\mathrm{amount}\;\mathrm{of}\;\mathrm{encapsulated}\;\mathrm{drug}\;\left(\mathrm{mg}\right)}{\mathrm{amount}\;\mathrm{of}\;\mathrm{total}\;\mathrm{lipid}\;\left(\mathrm{mg}\right)}$$

The entrapment efficiency (EE) is defined as the ratio of the amount of the drug encapsulated in the LNC relative to the total amount of the drug initially loaded [23]. The amount of encapsulated drug was quantified after the TFF dialysis system. LNC-Dox was broken down by dilution with EtOH and further analyzed using a UV–Vis spectrophotometer. The EE% was calculated as follows:

(2) $$\mathrm{EE}\%=\frac{\mathrm{amount}\;\mathrm{of}\;\mathrm{encapsulated}\;\mathrm{drug}\;\left(\mathrm{mg}\right)}{\mathrm{amount}\;\mathrm{of}\;\mathrm{loaded}\;\mathrm{drug}\;\left(\mathrm{mg}\right)}\times 100\%$$

### 2.7. Cryo-Electron Microscopy (Cryo-EM) of LNC-Dox

The structures of LNC-Dox were examined using an FEI Tecnai G2 F20 TWIN TEM (FEI, Hillsboro, OR, USA). Samples were prepared for cryo-EM on an FEI Vitrobot by pipetting 4 μL of the reaction mixture containing ~0.5 mM lipids onto a 200-mesh perforated carbon film (HC200-Cu, Electron Microscopy Sciences, Hatfield, PA, USA), blotted for 3 s, and stored in liquid nitrogen until imaging. Images were taken at a defocus value of ~1.8 μm under low-dose exposures (25–30 e/Å2) at 50,000-fold magnification on a FEI Tecnai F20, operating at 200 kV. All experiments were carried out at the Academia Sinica Cryo-EM Facility (Taipei, Taiwan).

### 2.8. In Vitro Drug Release and Normalized Drug Release Study of LNC-Dox

Drug release from liposomes was studied using a dialysis method. Experiments were carried out by placing LNC-Dox samples inside the Float-A-Lyzer^®^ (molecular weight cutoff 100 kDa), immersed in the release medium, at 37 °C and under 100 rpm stirring. Phosphate-buffered saline (PBS, pH 7.4) and citrate buffer (pH 5.6) were used for mimicking physiological conditions and the acidic tumor microenvironment, respectively [24]. Dissolution media aliquots (1 mL) were removed at 0.5, 1, 2, 4, 8, and 24 h time points and replaced with fresh release media. The amount of doxorubicin released at various times was determined by UV absorbance at 440 nm, and the concentration of the released doxorubicin was determined with the aid of a standard curve. Cumulative drug release percentage was calculated using the following formula:

(3) $$\mathrm{Drug}\;\mathrm{release}\;\left(\%\right)=\frac{\mathrm{release}\;\mathrm{drug}\;\mathrm{from}\;\mathrm{LNC}\;\left(\mathrm{mg}\right)}{\mathrm{total}\;\mathrm{drug}\;\mathrm{in}\;\mathrm{LNC}\;\left(\mathrm{mg}\right)}\times 100\%$$

In order to compare the acidic-triggered release properties of LNC-Dox, the drug release in acidic buffer was normalized against the cumulative drug release in PBS, with the aim to offer a clear view of identifying the extent of acidic-triggered release properties of LNC-Dox [25,26]. The normalized drug release was calculated by the following formula:

(4) $$\mathrm{Normalized}\;\mathrm{drug}\;\mathrm{release}=\frac{\mathrm{cumulative}\;\mathrm{drug}\;\mathrm{release}\;\mathrm{from}\;\mathrm{LNC}\;\mathrm{at}\;\mathrm{pH}\;5.6,24\;h\;\left(\mathrm{mg}\right)}{\mathrm{cumulative}\;\mathrm{drug}\;\mathrm{release}\;\mathrm{from}\;\mathrm{LNC}\;\mathrm{at}\;\mathrm{pH}\;7.4,\;24\;h\;\left(\mathrm{mg}\right)}$$

### 2.9. In Vitro Serum Stability

Briefly, the LNC-Dox was incubated with 20% fetal serum albumin (FBS) at 37 °C for up to 24 h [27,28]. At the indicated times, 50 μL of suspension was removed, and the released doxorubicin was collected by separation from LNC-Dox by passing through the spin column [29]. Time-dependent leakage of LNC-Dox was further quantified using the UV–Vis spectrophotometer.

### 2.10. In Vitro Cellular Study

C26 murine colon tumor cells were obtained from the Bioresource Collection and Research Center (BCRC) (Hsinchu, Taiwan). C26 cells were cultured in RPMI 1640 medium containing 10% fetal bovine serum (FBS) and 1% penicillin/streptomycin (Gibco, Grand Island, NY, USA), at 37 °C, in a humidified atmosphere of 5% CO_2_. The cellular uptake of LNC-Dox and Doxil^®^ was determined by seeding 5 × 10^5^ cells in a 6 cm dish and incubating them at 37 °C in 5% CO_2_ overnight. LNC-Dox or Doxil^®^ was added to each dish at a concentration of 50 μg/mL and incubated for 2 h. The cells were then washed with PBS twice, lysed with 5x lysis buffer for the determination of protein concentration, and the doxorubicin was extracted with acidic alcohol. The extracted doxorubicin was further quantified by measuring the fluorescence intensity with a spectrofluorometer (FluoroMax^®^-4, Horiba Jobin Yvon, Edison, NJ, USA).

The cytotoxicity of LNC-Dox was determined by MTT assay. Briefly, C26 cells were plated at a density of 5 × 10^4^ cells/mL in 96-well plates and incubated for 24 h. Cells were treated with different concentrations of LNC-Dox and Doxil^®^ for 2 h, and then replaced with drug-free culture medium and further incubated at 37 °C for 24 h. The medium was removed, and the cells were washed with PBS twice, and then incubated with MTT reagent for 1.5 h at 37 °C The amount of MTT formazan produced was analyzed at am absorbance of 570 nm with a DU 800 UV–Vis spectrophotometer (Beckman Coulter, Brea, CA, USA). The cells without LNC-Dox treatment were used as controls for 100% viability.

Doxorubicin accumulated in cell nuclei was determined as described in the report of Li et al. [30]. Briefly, C26 cells were seeded at a density of 3 × 10^5^ cells/mL in 6-well plate overnight. Cells were treated with LNC-Dox or Doxil^®^ for 2 h, and then replaced with drug-free culture medium and further incubated at 37 °C for 4 h. Cells were then washed with PBS and lysed in 0.5% (*w*/*v*) SDS. The cell lysates were then centrifuged at 800 rpm for 10 min. The supernatant was collected for the quantification of doxorubicin in the cytoplasm, and the pellet was resuspended and lysed with 5x lysis buffer to quantify the amount of doxorubicin in the nucleus. The accumulation of doxorubicin in the nucleus and cytoplasm was presented as the percentage associated with the total amount of internalized doxorubicin. 

### 2.11. Formulation Optimization by Response Surface Methodology (RSM)

To optimize the main effects, quadratic effects, and interaction effects of the formulation ingredients and process parameters, RSM combined with the BBD scheme was used in this study. BBD with three three-level factors—including the amount of DOPA, amount of DOPE, and FRR—was applied. Design-Expert 12 software was used to generate the 16 experimental runs, including 3 center-point trials, for further evaluation of the optimized LNC-Dox. The observed responses included particle size, polydispersity index (PDI), entrapment efficiency (EE%), drug-to-lipid ratio (D/L ratio), stability, and normalized drug release. The results obtained for each response were fitted to a second-order polynomial model used in the RSM, which is explained by Equation (5):

(5) $$y=A_{0}+A_{1}x_{1}+A_{2}x_{2}+A_{3}x_{3}+A_{4}x_{1}x_{2}+A_{5}x_{1}x_{3}+A_{6}x_{2}x_{3}+A_{7}x_{1}^{2}+A_{8}x_{2}^{2}+A_{9}x_{3}^{2}$$

where y represents the response variables, *A*_0_ is a constant, *A*_1_ to *A*_9_ are the regression coefficients, and *X*_1_, *X*_2_, and *X*_3_ are the independent factors. Analysis of variance (ANOVA) was used to examine the correctness and reliability of the models, including the coefficient of determination (R^2^), R-squared (adjusted), R-squared (predicted), and lack of fit (*p*-value). Moreover, 3D response surface graphs were generated to depict the influences of variable factors. The best possible formulation of LNC-Dox was selected and evaluated based on the results of RSM to achieve the optimal critical attributes of LNC-Dox. Meanwhile, the error percentages between the experimental and predicted values of critical attributes were examined for the accuracy and conformity of the models.

### 2.12. In Vivo Antitumor Efficacy of LNC-Dox

The in vivo antitumor efficacy against C26 murine colon tumor cells was determined in BALB/c mice obtained from the National Laboratory Animal Center (Taipei, Taiwan). The protocols were approved by the Institutional Animal Care and Use Committee (IACUC) of the National Taiwan University (IACUC Approval No: NTU-110-EL-00003). C26 cells (2 × 10^5^ cells) without a matrix were implanted into the right-side backs of mice (6–8 weeks of age) by subcutaneous injection. Mice (*n* = 5/group) were randomly arranged and as assigned to the control and the experimental groups. The free form-doxorubicin, liposomal Dox, and LNC-Dox were diluted with 0.9% NaCl. The drug concentration for intravenous injection was 5 mg/kg via the tail vein when the tumor grew to 100 mm^3^ in volume. In the control group, 0.9% NaCl was given. The tumor size and body weight of the mice were monitored every 2 days. Tumor volume of 2500 mm^3^ was considered as the end point, upon which the mice would be euthanatized by CO_2_ inhalation.

### 2.13. Statistics

Data values are represented as the mean ± standard deviation from at least three independent experiments. The statistic difference in tumor volume between groups was analyzed by two-way ANOVA. Kaplan–Meier survival curves were used to analyze the survival rate, and the log-rank test was performed to analyze the differences. A *p*-value less than 0.05 was considered statistically significant.

## 3. Results and Discussions 

### 3.1. Influence of Total Flow Rate and Flow Rate Ratio on LNC-Dox Formation 

**Figure 1:**
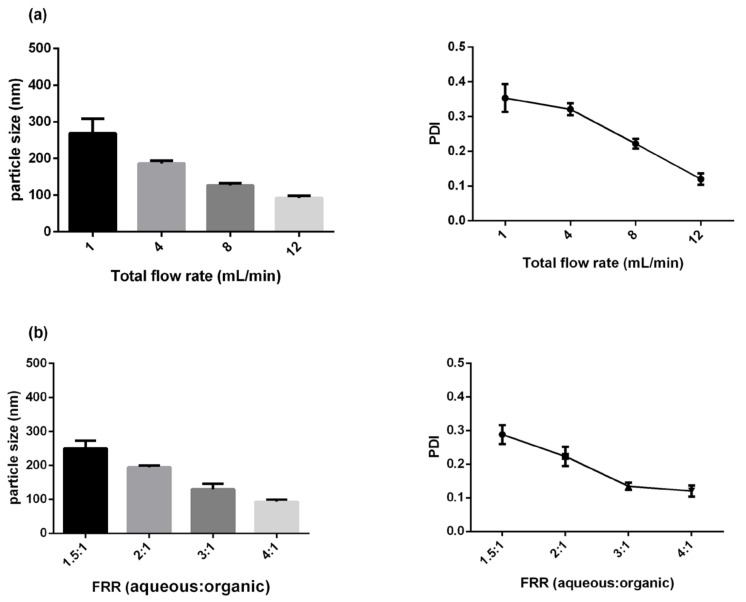
Characteristics of LNC-Dox: (**a**) Effects of TFR on particle size and PDI at a fixed FRR (4:1). (**b**) Effects of FRR on particle size and PDI at a fixed TFR (12 mL/min). Results are the mean ± SD obtained from three independent experiments (*n* = 3).

It is known that the parameters of microfluidic rapid mixing—such as total flow rate (TFR) and flow rate ratio (FRR)—have significant effects on the physicochemical properties of nanocarrier formation [31,32]. Therefore, we began to study the effects of TFR on the fabrication of Dox-loaded nanocarriers (LNC-Dox) at a fixed FRR (4:1). The lipid composition of LNC-Dox contains HSPC, cholesterol, DSPE-PEG2000, and anionic phospholipid EPG at the molar ratio of 9.4:41.9:1.9:46.8. Negatively charged phospholipid EPG was used to increase the association between carriers and doxorubicin, aiming to increase the drug-loading capacity of the nanocarriers. Figure 1a shows the effects of TFR on the particle size and PDI of LNC-Dox. The size of LNC-Dox ranged from 269.2 to 92 nm at varying speeds from 1 to 12 mL/min. LNC-Dox produced with a lower TFR showed larger particle size (left-hand panel of Figure 1a) and higher PDI values (right-hand panel of Figure 1a), indicating a non-homogeneous size distribution of LNC-Dox. These results might have been related to the low TFR, which increases the chance of inadequate lipid aggregation during the microfluidic mixing process. Therefore, a high TFR of 12 mL/min was used for further LNC-Dox production. On the other hand, FRR correlates with the changing polarity inside the microfluidic channels. High FRR could rapidly increase the polarity of the aqueous phase and the organic phase of the mixture. As shown in Figure 1b, small LNC-Dox with high homogeneity (PDI < 0.2) can be produced at a high FRR of 4:1, due to more rapid mixing and the increase in dilution effects. This result could be supported by the proposed mechanism of nanoparticles’ formation via the precipitation method—that smaller nanoparticles could be produced if the mixing intensities are high enough [33,34]. High mixing intensities could be achieved when the mixing time was much shorter than the aggregation time. In this case, the solution became supersaturated, and the nucleation events dominated the particles’ formation to produce smaller particles. Based on these results, LNC-Dox was produced by microfluidic rapid mixing at a TFR of 12 mL/min and FRR of 4:1 in the following experiments.

### 3.2. LNC-Dox Made with EPG Showed a High D/L Ratio and Exhibited an Electron-Dense Core Structure Revealed by Cryo-TEM

**Figure 2:**
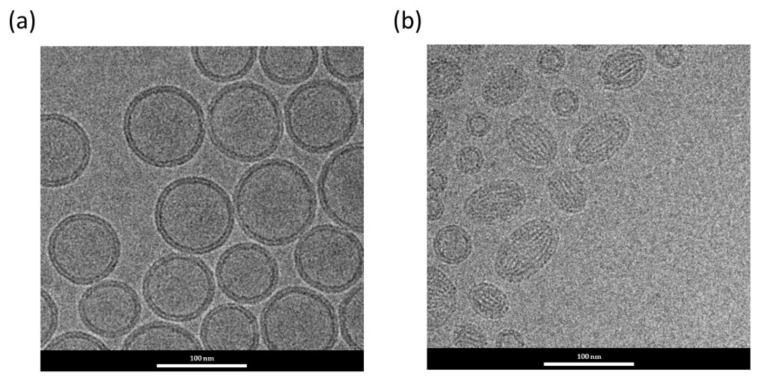
Cryo-TEM micrograph of Dox-loaded lipid-based nanocarriers prepared without (**a**) or with (**b**) EPG.

As mentioned above, EPG was used in the present formula to increase the association between doxorubicin and the carriers. Therefore, we further examined the possible differences in the characteristics and morphological structure of Dox-loaded nanocarriers prepared with or without EPG. The results of dynamic light scattering and D/L ratio are shown in Table 1. Dox-loaded nanocarriers prepared in the absence of EPG showed a low D/L ratio (0.062) compared to that of Dox-loaded LNCs containing EPG (0.225). The electrostatic interaction between cationic doxorubicin and anionic phospholipid EPG could be the main cause of the increased drug encapsulation during the LNC formation process. In fact, the D/L ratio of LNC-Dox developed in this study was also significantly higher than that in published reports using anionic lipids for Dox-loaded liposomes prepared via traditional methods (D/L ratio < 0.06) [35,36,37,38]. This might have been due to our bottom-up fabrication using microfluidic technology, which allowed the full interaction between EPG and doxorubicin during the control of the mixing process. It is likely that drugs and charged lipids could arrange well at the interface of the aqueous and organic phases due to the laminar flow created by rapid mixing inside the microfluidic channels. In fact, the complexed interaction between doxorubicin and EPG resulted in a different morphological structure of the nanocarriers, as shown in the cryo-TEM image in Figure 2. Dox-loaded lipid nanocarriers without EPG exhibited the traditional bilayer vesicles of liposomes prepared via microfluidics (Figure 2a). However, in the presence of EPG, complexes of doxorubicin and lipids were found in the structured core of LNC-Dox (Figure 2b), which was similar to that of the lipid-based nanoparticles encapsulated with RNA prepared by microfluidics [39,40]. The structured core might have been contributed to by the inverted structures, such as the hexagonal H_II_ phase or inverted micelles, in the mixture of doxorubicin and anionic lipid EPG. 

### 3.3. In Vitro Characterization and In Vivo Therapeutic Efficacy of LNC-Dox 

**Figure 3:**
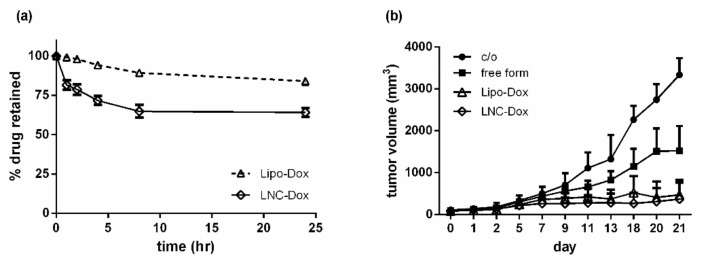
(**a**) In vitro serum stability of Lipo-Dox and LNC-Dox over a time period of 24 h. Data are expressed as the mean ± SD (*n* = 3). (**b**) In vivo therapeutic efficacy of different doxorubicin formulations. Mice were intravenously administrated with indicated drugs (10 mg/kg) three times (day 0, day 7, and day 14) during the treatment. Data are expressed as the mean ± SD (*n* = 5).

**Figure 4:**
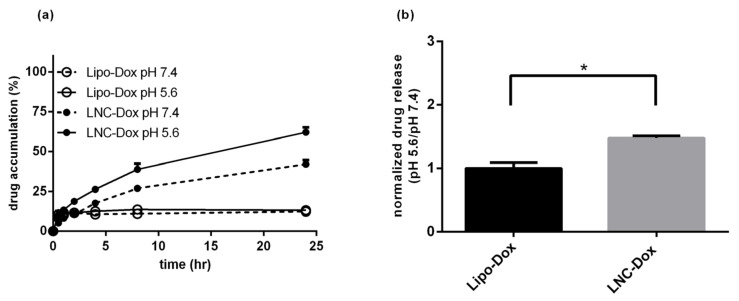
(**a**) In vitro drug release profiles of LNC-Dox and Lipo-Dox were performed in PBS (pH 7.4) and citrate buffer (pH 5.6) over a time period of 24 h. (**b**) The normalized drug release of LNC-Dox and Lipo-Dox (* *p* < 0.001). Data are expressed as the mean ± SD (*n* = 3).

Figure 3a shows the serum stability of LNC-Dox fabricated via microfluidics and liposomal doxorubicin (Lipo-Dox) prepared via the traditional method of an ammonium sulfate gradient. About 64.2% and 80.8% of the Dox still remained inside the LNCs and liposomes, respectively, after 24 h incubation with serum protein. To evaluate the antitumor effect of LNC-Dox, an in vivo therapeutic study was performed in a C26 syngeneic BALB/c tumor model. As shown in Figure 3b, the tumors became larger in the saline control and free-from doxorubicin treatment groups. However, significant tumor reduction was found in mice treated with Lipo-Dox and LNC-Dox. Although the serum stability of LNC-Dox was less than that of Lipo-Dox, the therapeutic efficacy showed no significant difference between these two Dox formulations. This might have resulted from the preferred release properties of LNC-Dox within the acidic tumor microenvironment, as depicted in Figure 4. As shown in Figure 4a, the accumulated doxorubicin released from LNCs and liposomes was about 32.5% and 19.2%, respectively, during the first 24 h at pH 7.4. Meanwhile, in the acidic environment, the accumulated drug was about 54.7% and 12.54% from LNC-Dox and Lipo-Dox, respectively. To further compare the acidic-preferred release properties of Lipo-Dox and LNC-Dox, cumulative drug released in citrate buffer was normalized against that of PBS. As shown in Figure 4b, the normalized drug release of LNC-Dox was 1.5 times higher than that of Lipo-Dox, suggesting the preferred release of LNC-Dox in an acidic tumor microenvironment. We then further assessed whether the improved serum stability of this LNC-Dox could significantly enhance the therapeutic efficacy. To this end, the normalized drug release was studied as the critical attribute of LNC-Dox for the following formulation optimization. 

### 3.4. Modification of LNC-Dox Lipid Composition Based on the Critical Packing Parameter

**Figure 5:**
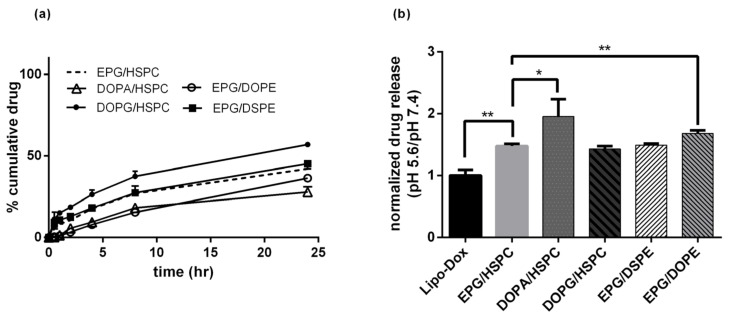
(**a**) In vitro drug release of LNC-Dox with different lipid compositions in PBS. (**b**) Normalized drug release of LNC-Dox with different lipid compositions (* *p* < 0.05; ** *p* < 0.01). Data are expressed as the mean ± SD (*n* = 3).

Previous studies have shown that RNA-encapsulated lipid nanoparticles exhibit an electron-dense core, which might consist of reverse micelles or inverse hexagonal structures formed by the electrostatic interaction between anionic nucleic acids and ionizable lipids [41]. As shown in Figure 2b, a similar electron-dense core was also found in LNC-Dox. In this regard, one approach to increase the stability of LNC-Dox is to further increase the stability of the internal structure of the drug carriers. The critical packing parameter (CPP) is a theoretical number that could be used to determine the type of aggregation formed by amphiphiles [42,43]. It has been indicated that a CPP higher than 1 tends to form an inverse structure [44]. We thus hypothesized that phospholipids with a higher CPP value might result in the formation of a more stable internal structure of LNC-Dox. The present lipid composition of LNC-Dox contained HSPC, cholesterol, DSPE-PEG2000, and anionic phospholipid EPG. For the further optimization study, the anionic phospholipids DOPG and DOPA were used to replace the EPG, while the neutral phospholipid HSPC was replaced by DSPE and DOPE. The stability of Dox-loaded nanocarriers was examined by in vitro release studies in PBS (pH 7.4). As shown in Figure 5a, when EPG was replaced with DOPA, the cumulative drug release was reduced to approximately 24.3% over 24 h, indicating an increase in the stability of LNC-Dox. Furthermore, replacement of HSPC with DOPE could also stabilize the LNC-Dox with the accumulative drug release in PBS (approximately 28.1%). Figure 5b shows the normalized drug release of LNC-Dox with different lipid compositions. LNC-Dox composed of DOPA or DOPE showed higher normalized drug release compared to other groups. These results indicate that the stability of LNC-Dox could be improved by including phospholipids with higher CPP values, favoring the formation of an inverted structure, while maintaining the preferred release properties in acidic conditions. Therefore, DOPA and DOPE were included in the formulation of LNC-Dox for the subsequent optimization study. To this end, response surface methodology (RSM) was used to determine the best possible formulation composition. 

### 3.5. LNC-Dox Optimization by Response Surface Methodology (RSM)

As shown in Table 2, three factors—including the amount of DOPA, the amount of DOPE, and the FRR—were used in the RSM optimization process. The responses included particle size, PDI, entrapment efficiency, D/L ratio, stability, and the normalized drug release, which were chosen based on the critical quality attribute (CQA) of LNC-Dox. The obtained responses of the experimental trials are summarized in the Supplementary Materials (Table S1). The results obtained for each response were further fitted to the response surface models, including a linear regression model, 2F1 (sequential sum of squares for the two-factor interaction) model, and quadratic models (Supplementary Materials, Table S2). According to the sequential *p*-value, a quadratic model was suggested for studying the stability, while the linear models were proposed for analyzing particle size, D/L ratio, and normalized drug release. Meanwhile, a 2F1 model was carried out to examine the entrapment efficiency. As shown in Table 3, the differences between the adjusted and predicted R-squared values were within 0.2, further verifying the correctness of these models. ANOVA analysis showed the statistical significance of these models (Supplementary Materials, Tables S3–S7). The diagnostic plots for validating the obtained models further showed that the residuals were found to cluster around an approximately straight line, indicating the normal distribution of the residuals (Supplementary Materials, panel a of Figures S1–S6). Moreover, the graphs of residuals versus predicted and residuals versus runs showed that the residuals had randomly scattered within the standard deviation range, indicating that the errors were independent and there was no need to repeat any of the runs (Supplementary Materials, panel b of Figures S1–S6). 

#### 3.5.1. The Effects of Lipid Composition on Particle Size Distribution

**Figure 6:**
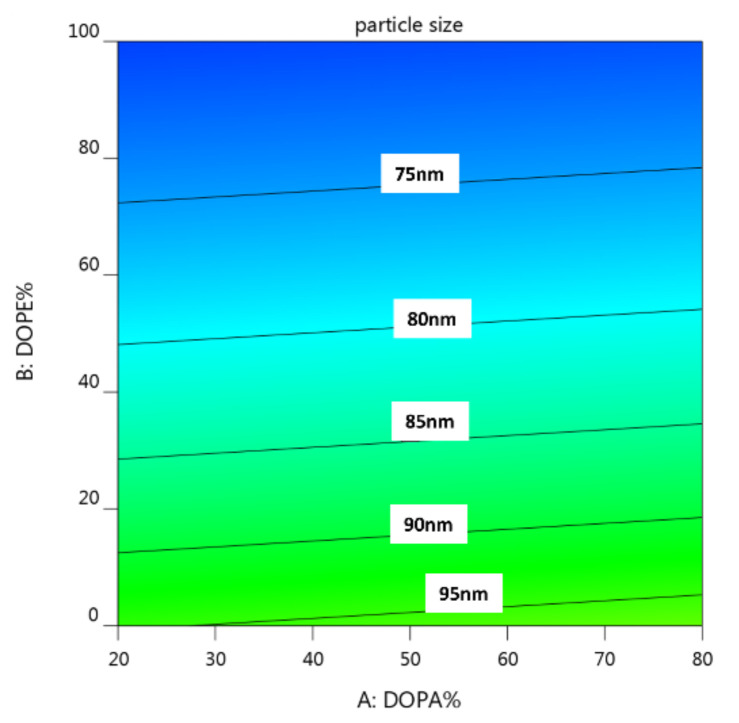
Contour plot for the interaction of lipid composition with particle size.

As shown in the contour plot of Figure 6, the design points of particle size were all smaller than 200 nm, which was within the range of CQA set for LNP-Dox (Table 2). These results indicated that the basic formulation, including the ratio of lipid composition and the process parameters used in this study, was suitable for forming Dox-loaded nanosized particles. Interesting, the particle size became smaller with the increasing amounts of DOPE (Figure 6). However, the particle size of LNC-Dox was not significantly affected by the amount of DOPA. The different impacts of DOPE and DOPA on the particle size might be a result of the different roles of these two lipids in forming the LNC-Dox. The anionic phospholipids DOPA and EPG are nucleating agents used to interact with the cationic drug doxorubicin, and further form the inverted structures of lipid nanocarriers. On the other hand, the neutral phospholipids DOPE and HSPC are used to form the monolayer that covers the inverted structure. Although the increased DOPA could attract more doxorubicin to form inverted structures, the electrostatic repulsion between anionic phospholipids might also have loosened the core, and further enlarged and destabilized the structure of LNC-Dox. We suspected that DOPE could be inserted inside the core, thereby helping the formation of the inverted core structure during the rapid mixing. In this regard, the increased insertion of DOPE could reduce the repulsion between charged lipids, resulting in more tightly packed and smaller lipid nanoparticles. This could explain the relationship of the particle size of LNC-Dox with the amounts of neutral phospholipids.

#### 3.5.2. The Effects of Lipid Composition and FRR on PDI

**Figure 7:**
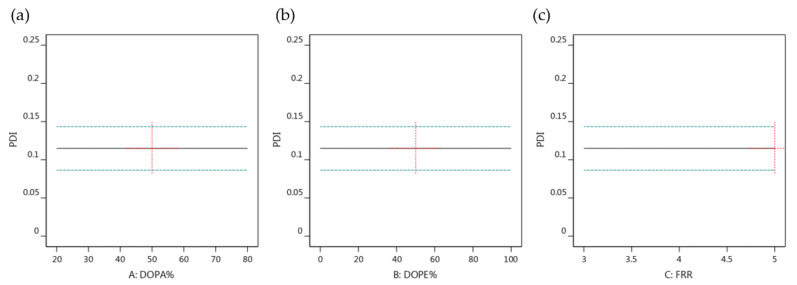
One-factor effect of (**a**) amount of DOPA, (**b**) amount of DOPE and (**c**) FRR on particle size.

As shown in the Supplementary Materials (Table S2), except for the mean model, the sequential *p*-values of the other polynomial models were insignificant. Table 3 further shows that the fit statistic is a negative predicted R^2^ for the RSM model, implying that the overall mean might be a better predictor of the response. In addition, the changes in the lipid composition and the FRR had insignificant effects on the PDI value, as shown in Figure 7. All of these results suggest that the PDI value of LNC-Dox was predetermined by the formulation parameters set at the beginning of the preformulation selection.

#### 3.5.3. The Effects of DOPA Amounts on the EE% and D/L Ratio of LNC-Dox

**Figure 8:**
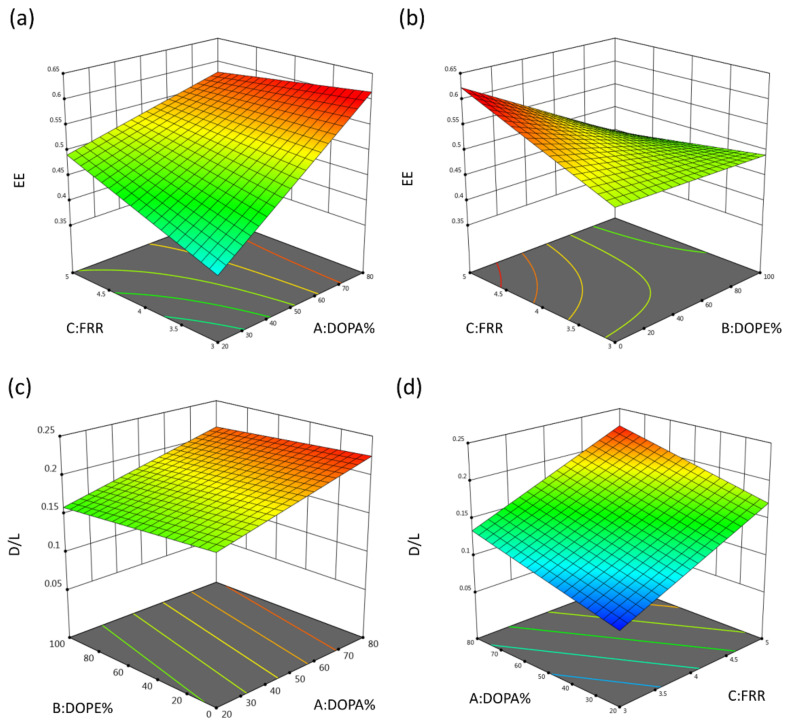
3D surface plot for (**a**) interaction of DOPA% and FRR with EE%, (**b**) interaction of DOPE% and FRR with EE%, (**c**) interaction of DOPA% and DOPE% with D/L ratio, and (**d**) interaction of DOPA% and FRR with D/L ratio.

Based on the function of DOPA%, DOPE%, and FRR (Table 3), the 3D surface plots were generated to model the D/L ratio. As shown in Figure 8a,c, the EE% and D/L of LNC-Dox increased with the increase in the DOPA ratio, which was possibly due to the higher affinity of DOPA for doxorubicin compared to that between EPG and doxorubicin. This argument could be supported by the report that DOPA showed higher binding affinity for doxorubicin than other anionic phospholipids [45]. Dewolf et al. reported that the preferred binding of doxorubicin with DOPA was related not only to its electrostatic interaction, but also to the stacking of free-form doxorubicin onto the formed Dox–DOPA complex. In addition, during microfluidic rapid mixing, the microchannel offered more opportunities for the interaction of doxorubicin and DOPA by creating the high surface-to-volume ratio. Therefore, Dox and DOPA aligned properly at the interface between the aqueous and organic solutions, leading to good and tight binding. Combined together, the EE% and D/L ratio could be higher when DOPA was included in the LNC-Dox formulation, as shown in Figure 8a,c.

During microfluidic processing, the FRR could influence the solution’s polarity, which might have affected the drug-loading capacity in forming the nanocarriers. Therefore, we addressed the impact of FRR on the EE and D/L ratio of LNC-Dox; as shown in Figure 8a,b, higher EE% was correlated with higher FRR. This might have been related to the mixing rate of the aqueous and lipid phases, which was accelerated by high FRR during the microfluidic mixing process as a result of two mechanisms [46]: First, high FRR would elevate the difference in fluid velocity between the aqueous and organic phases, which further reduced the effective diffusion of these two phases. The other mechanism was that the increase in FRR would reduce the amounts of organic phase, thereby further decreasing the width of the organic stream inside the mixing channel of the microfluidic chip and reducing the thickness of the diffusion layer. Therefore, the mixing rate of these two phases increased at high FRR, which accelerated the polarity increase in the lipid phase [46]. To clarify, the concentration of ethanol decreased rapidly, resulting in the reduction in the improper aggregation that ensured the good complexation of anionic phospholipids and doxorubicin. Furthermore, the decrease in DOPE% amplified the effects of FRR on EE%, as shown in Figure 8b. This is because too much DOPE can have a negative influence on the LNC formation, thereby lowering the EE%, which could not be rescued by elevating the FRR. In fact, the positive effect of FRR on EE% was correlated with the influence of the FRR on the D/L ratio, as shown in Figure 8d. Taken together, these results demonstrate that the high FRR could accelerate the polarity increase of the organic phase by ensuring good complexation between the anionic phospholipids and doxorubicin. 

#### 3.5.4. The Effects of Lipid Composition on the Stability and Normalized Drug Release of LNC-Dox

**Figure 9:**
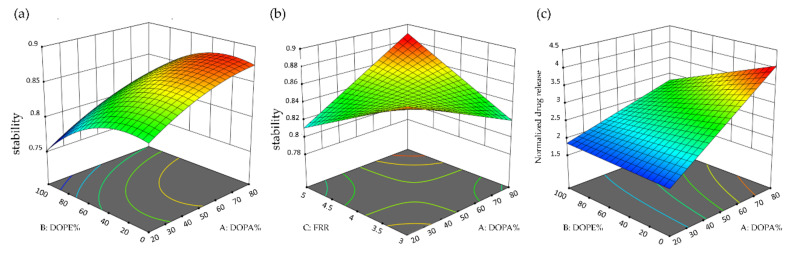
3D surface plot for (**a**) the interaction of DOPA% and DOPE% with stability, (**b**) the interaction of DOPA% and FRR with stability, and (**c**) the interaction of DOPA% and DOPE% with normalized drug release.

Figure 9a,b show the 3D surface plots of LNC-Dox stability based on the function of DOPA%, DOPE%, and FRR. The stability increased according to the percentage of DOPA, which was correlated with the tight packing of doxorubicin and DOPA by electrostatic interaction and other hydrophobic interactions, as mentioned in Section 3.5.3. The good complexing between DOPA and doxorubicin further stabilized the core of LNC-Dox. As shown in Figure 9a, the stability of LNC-Dox significantly decreased when DOPE% was set to more than 40%. This could be explained by the function of neutral lipids in the formation of LNC-Dox. The neutral lipids may act as the excipient for forming the monolayer outside the complex of anionic lipids and the drug, and could also be used to fill in the gaps and spaces in the lipid–drug complex. However, DOPE with a large CPP value preferentially forms a reverted structure that tends to fill the gaps inside, rather than forming a planar structure outside the LNC. Therefore, with an excessive amount of DOPE, the other neutral lipid (HSPC) might not be enough to form the outer monolayer of LNC-Dox. This could possibly result in the incomplete formation of LNC-Dox, leading to lower stability. 

Additionally, both factors—DOPA% and DOPE%—were found to be significant in explaining the response of normalized drug release, as shown in the Supplementary Materials (Table S7). 3D surface plots were further generated to outline the effects of DOPA% and DOPE% on the normalized drug release (Figure 9c). The normalized drug release was calculated as an index for the extent of the preferred release of doxorubicin from LNC-Dox in an acidic environment. The results demonstrated that the increase in the normalized drug release was correlated with the increase in DOPA. In addition, the effect of DOPE on normalized drug release became more significant when DOPA% was high, possibly relating to the preferred release in acidic conditions. It has been reported that, at low pH, protons compete with doxorubicin for binding to DOPA, leading to more doxorubicin release [45]. Therefore, with the increase in proton concentration in an acidic environment, the interaction between DOPA and doxorubicin decreases, and resulting in the increased release of doxorubicin from LNCs. This explains why LNC-Dox showed high normalized drug release in the presence of high DOPA% (Figure 9c). However, the increase in normalized drug release would be compromised by the increasing amounts of DOPE, which might relate to the decreased stability of LNC-Dox due to the excess amount of DOPE, as discussed above. Taken together, these results indicate that a large amount of DOPA should be included, while the amount of DOPE should be limited in a minimum range, for high normalized drug release.

### 3.6. Best Possible Formulation Selection and the Validation of Models 

**Figure 10:**
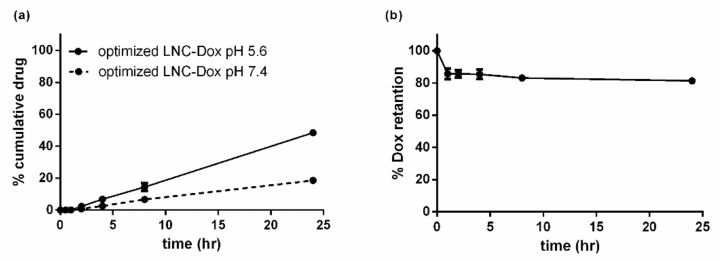
In vitro characterization of the optimized LNC-Dox: (**a**) In vitro drug release of the optimized LNC-Dox. (**b**) Serum stability of the optimized LNC-Dox. Data are expressed as the mean ± SD (*n* = 3).

Based on the above optimization studies, RSM with a BBD scheme provided the best possible formulation, which achieved the desired responses, including the maximum stability, EE%, D/L ratio, and normalized drug release. The best possible formulation for LNC-Dox was composed of DOPA:EPG:DOPE:HSPC:cholesterol:DSPE-PEG2000 at a molar ratio of 37.44:9.36:0.47:8.93:41.9:1.9, and the FRR was set to 5:1. The prepared LNC-Dox was tested, and the experimental responses were further compared to the predicted values to validate the accuracy. As shown in Table 4, the corresponding error percentage between the predicted and actual values was lower than 20%, except for the normalized drug release. As mentioned above, the normalized drug release was associated with the complexation of doxorubicin and the anionic phospholipid DOPA. Despite the large amount of H^+^ in the acidic environment, the tight complexation was not easily reversed, resulting in a higher error percentage between the actual and predicted values of the normalized drug release. However, the normalized drug release was still elevated to 3.15 (Table 4) compared to that of the unoptimized formulation (1.5, shown in Figure 4b). In fact, the stability of the optimized LNC-Dox increased to 83.45% (Figure 10a), as compared to 66.4% (Figure 4a) for the unoptimized formulation. Meanwhile, the serum stability also increased to about 82% (Figure 10b). These results indicate that this LNC formula could be a good nanocarrier for doxorubicin delivery with a high D/L ratio and acidic release properties. 

### 3.7. In Vitro and In Vivo Therapeutic Efficacy of the Optimized LNC-Dox 

**Figure 11:**
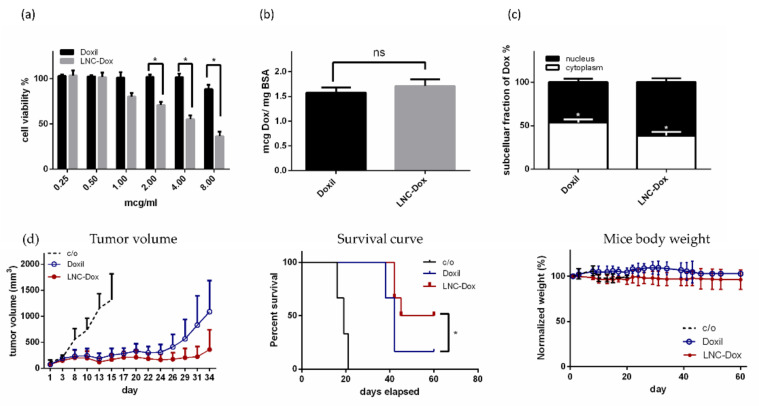
In vitro and in vivo therapeutic efficacy of the optimized LNC-Dox against murine C26 tumor cells: (**a**) In vitro cytotoxicity of Doxil^®^ and LNC-Dox (* *p* < 0.01). (**b**) Cellular uptake of Doxil^®^ and LNC-Dox. (**c**) Intracellular distribution of doxorubicin in C26 cells treated with Doxil^®^ and LNC-Dox (* *p* < 0.01). (**d**) Left: effects of Doxil^®^ and the optimized LNC-Dox on tumor progression (* *p* < 0.01). Middle: Kaplan–Meier analysis of survival of tumor-bearing mice treated with Doxil^®^ and the optimized LNC-Dox (* *p* < 0.05); survival times reflect the times at which mice were euthanatized due to the tumor size exceeding 2500 mm^3^. Right: measurement of body weight for the tumor-bearing mice treated with saline, Doxil^®^, and the optimized LNC-Dox. Mice were intravenously administrated with the indicated drugs (10 mg/kg) twice (day 0 and day 7) during the treatment. Data are expressed as the mean ± SD for each group (*n* = 6).

To further evaluate the therapeutic efficacy of optimized LNC-Dox composed of EPG, DOPA, cholesterol, HSPC, DOPE, and DSPE-mPEG2000, we first examined its in vitro cytotoxicity against murine C26 tumor cells. As shown in Figure 11a, the optimized LNC-Dox showed higher cytotoxicity to C26 cells compared to Doxil^®^. However, there was no significant difference in the cellular uptake between these two different doxorubicin-loaded lipid nanocarriers (Figure 11b). As reported, the cytotoxic action of doxorubicin takes place in the nucleus by inhibiting topoisomerase II. In this regard, doxorubicin must be released from the nanocarrier after cellular uptake and entry into the nucleus to exert its cytotoxic effect. We hypothesized that the higher cytotoxicity of LNP-Dox might be related to its better endosomal escape ability due to the property of acidic-preferred release, resulting in more doxorubicin being accumulated in the nucleus. In fact, as shown in Figure 11c, the optimized LNC-Dox exhibited higher nucleic accumulation of doxorubicin compared to Doxil^®^. These results indicate that the preferred release of LNC-Dox in acidic environments could enhance the intracellular drug release and enter the nucleus to exert better therapeutic efficacy.

The in vivo therapeutic efficacy of optimized LNC-Dox was further examined in BALB/c mice bearing C26 tumors. As shown in the left panel of Figure 11d, the optimized LNC-Dox showed better tumor regression compared to the commercial drug Doxil^®^. In fact, the overall survival rate was also significantly improved in mice treated with the optimized LNC-Dox compared to that those treated with Doxil^®^ (middle panel of Figure 11d). Moreover, no significant changes in body weight were found in the mice treated with LNC-Dox, suggesting the safety of using this LNC-Dox (right panel of Figure 11d). These results clearly indicate the advantages of this optimized LNC-Dox over Doxil^®^ in terms of suppressing tumor growth. Doxorubicin encapsulated in PEGylated liposomes is released both in the tumor’s extracellular fluid, and intracellularly via the endosomal escape process [47]. However, the slow drug release and poor endosomal escape capability of PEGylated liposomes in fact impedes their therapeutic efficacy [48]. As shown here, the present Dox-loaded LNCs fabricated by microfluidics have preferred drug release in acidic environments, and better therapeutic efficacy in mice bearing C26 tumors. In the future, therapeutic efficacy and pharmacokinetic studies will need to be carried out in human tumor xenografts. 

## 4. Conclusions

In this study, Dox-loaded lipid-based nanocarriers composed of neutral and anionic phospholipids were developed and fabricated by microfluidic rapid mixing. The optimized LNC-Dox showed better tumor regression and overall survival rates compared to the commercial drug Doxil^®^. There are two advantages of using this LNC-Dox for tumor treatment: First, doxorubicin encapsulated in this LNC delivery system had a high D/L ratio, which could offer a higher therapeutic dose without excessive use of lipids for clinical development. Second, the preferred release in acidic environments could increase the amounts of bioavailable drug for tumor treatment. Therefore, the strategy used in the present formulation’s development, combined with the bottom-up microfluidic technique, showed its potential in improving drug-loading capacity and simplifying the manufacturing processes for the translational development of lipid-based nanocarrier drugs. 

## 5. Patents

The work reported in this manuscript has a patent pending under the title of composition and method of preparation for lipid formulations comprising charged lipids.

## Figures and Tables

**Table 1:** Characteristics of Dox-loaded lipid-based nanocarriers produced with or without EPG. Data are expressed as the mean ± SD (*n* = 3).

Sample	Dox-Loaded Lipid-Based Nanocarriers	
	Without EPG	With EPG
Particle size (nm)	86.3 ± 21.8	85.4 ± 22.1
PDI	0.051	0.165
D/L (*w*/*w*)	0.062	0.225

**Table 2:** Factors and responses used in Box–Behnken design for optimization.

Factors		Levels		
		−1	0	1
A	DOPA%	20	50	80
B	DOPE%	0	50	100
C	FRR	3	4	5
*Responses*		*Goals*		
A	Particle size	<200 nm		
B	PDI	<0.3		
C	Entrapment efficiency	>40%		
D	D/L ratio	>0.125		
E	Stability	>56% (within 24 h)		
F	Normalized drug release	>1.5		

**Table 3:** Summary of regression analysis of models.

Response	Suggested	Model Equations in Terms of Important Coded Factors	Model Sequential *p*-Value	R^2^	Adjusted R^2^	Predicted R^2^	Adequate Precision
Particle size	Linear	−0.0000005672 × A + 0.000009104 × B − 0.000001001 × C	<0.001	0.9593	0.9742	0.8746	21.608
PDI	Mean	+0.1149	<0.0001	0	0	−0.1378	N/A
EE	2F1	+0.5153 + 0.0811 × A − 0.0454 × B + 0.0200 × C − 0.0382 × AC − 0.0421 × BC	0.0003	0.9761	0.9641	0.9289	30.0192
D/L ratio	Linear	+0.1530 + 0.0269 × A + 0.0454 × C	<0.0001	0.9422	0.9333	0.9178	32.559
Stability	Quadratic	+0.8486 + 0.0058 × A − 0.0218 × B + 0.0014 × C + 0.0333 × AC − 0.0265 × B^2^	0.0024	0.9582	0.9372	0.8570	21.9604
Normalized drug release	Linear	+2.70 + 0.5558 × A − 0.3800 × B − 0.0777 × C − 0.3292 × AB + 0.3340 × AC	<0.0001	0.9695	0.9542	0.9075	30.4475

**Table 4:** Predicted and actual values of the optimized LNC-Dox. Values are expressed as the mean ± SD (*n* = 3).

Response	Predicted Value	Actual Value	Error Percentage %
Particle size (nm)	95.139	97.5 ± 22.6	2.48
PDI	0.115	0.134 ± 0.016	16.5
EE%	65.9	62.7 ± 3.9	4.8
D/L	0.224	0.225 ± 0.027	0.4
Normalized drug release	3.999	3.15 ± 0.11	21.2
Stability	0.877	0.8345 ± 0.018	4.8

## Supplementary Materials

The following are available online at https://www.mdpi.com/article/10.3390/biomedicines10061259/s1, Figure S1: The diagnostic plots for the validation of obtained model for particle size: (a) normal plot of residuals, (b) residuals versus predicted and (c) residuals versus run order, Figure S2: The diagnostic plots for the validation of obtained model for PDI: (a) normal plot of residuals, (b) residuals versus predicted and (c) residuals versus run order. Figure S3: The diagnostic plots for validation of obtained model for EE%: (a) normal plot of residuals, (b) residuals versus predicted and (c) residuals versus run order, Figure S4: The diagnostic plots for the validation of obtained model for D/L ratio: (a) normal plot of residuals, (b) residuals versus predicted and (c) residuals versus run order, Figure S5: The diagnostic plots for the validation of obtained model for stability: (a) normal plot of residuals, (b) residuals versus predicted and (c) residuals versus run order, Figure S6: The diagnostic plots for the validation of obtained model for normalized drug release: (a) normal plot of residuals, (b) residuals versus predicted and (c) residuals versus run order, Table S1: BBD for the study of four experimental factors with experimental results, Table S2: Fit summary of the models, Table S3: ANOVA for particle size, Table S4: ANOVA for EE%, Table S5: ANOVA for D/L ratio, Table S6: ANOVA for stability, Table S7: ANOVA for normalized drug release.
