# Supplementary material for: Fabrication of Doxorubicin-Loaded Lipid-Based Nanocarriers by Microfluidic Rapid Mixing
Source: Biomedicines. 2022 May 27;10(6):1259. doi: 10.3390/biomedicines10061259 (PMC9219747; doi:10.3390/biomedicines10061259)
Supplement: Supplementary file 1 [file biomedicines-10-01259-s001.zip › biomedicines-1736715-supplementary.pdf]

Supplementary data

Authors: Chia Ying Lee, Tsuimin Tsai, Po-Chun Peng, and Chin-Tin Chen

Contact: chintin@ntu.edu.tw

Supplementary Table S1. BBD for the study of four experimental factors with experimental results.

	Factor A	***Factor B***	***Factor C***	***Response 1***	***Response 2***	***Response 3***	***Response 4***	***Response 5***	***Response 6***
Run	DOPA%	DOPE%	FRR	particle size (nm)	PDI	EE%	D/L	normalized drug release	stability
1	80	50	3	81.3	0.036	0.608209	0.141833	3.02619	0.821587
2	50	50	4	85.9	0.189	0.518791	0.155574	2.85914	0.840741
3	80	50	5	76.8	0.11	0.569392	0.228171	3.40947	0.883318
4	20	0	4	97.3	0.091	0.465974	0.124389	2.09012	0.832157
5	50	100	5	75.5	0.134	0.432196	0.183374	2.35118	0.806795
6	50	0	3	88.3	0.081	0.503435	0.1015	3.09142	0.835053
7	20	50	5	78.9	0.088	0.493326	0.172891	1.70435	0.812254
8	50	50	4	76.5	0.131	0.503531	0.139798	2.82558	0.846326
9	50	100	3	71.9	0.15	0.474224	0.11429	2.31573	0.804038
10	20	50	3	82.3	0.113	0.379294	0.0787515	2.65695	0.883535
11	50	50	4	68.2	0.245	0.516125	0.168992	2.8214	0.85288
12	20	100	4	67.2	0.079	0.402263	0.126315	1.94249	0.787998
13	80	100	4	73.9	0.088	0.569696	0.183753	2.47003	0.802138
14	80	0	4	94.5	0.122	0.64243	0.163955	3.93458	0.855289
15	50	50	4	73.7	0.033	0.536717	0.150196	2.65684	0.848149
16	50	0	5	117.7	0.148	0.629947	0.215002	3.00351	0.853063

Supplementary Table S2. Fit summary of the models.

Response	Source	Sequential p-value	Lack of Fit p-value	
Particle size	**Linear**	**0.0009**	**0.8404**	**suggested**
	2FI	0.8008	0.7460	
	Quadratic	0.4987	0.7199	
PDI	**Mean**	**< 0.0001**	**NA**	**suggested**
	Linear	0.9441	0.9627	
	2FI	0.7932	0.9260	
	Quadratic	0.5563	0.9561	
EE	Linear	< 0.0001	0.0517	
	**2FI**	**0.0003**	**0.4361**	**suggested**
	Quadratic	0.8116	0.2692	
D/L ratio	**Linear**	**< 0.0001**	**0.7050**	**suggested**
	2FI	0.1496	0.8562	
	Quadratic	0.8897	0.6602	
Stability	Linear	0.1513	0.0073	
	2FI	0.0414	0.0152	
	**Quadratic**	**0.0024**	**0.1656**	**suggested**
Normalized drug release	**Linear**	**< 0.0001**	**0.7050**	**suggested**
	2FI	0.1496	0.8562	
	Quadratic	0.8897	0.6602	

Supplementary Table S3. ANOVA for particle size.

Source	*Sum of squares*	*df*	*Mean square*	*F-value*	*p-value*	
**Model**	6.736E-10	3	2.245E-10	7.74	0.0009	significant
A-DOPA%	2.574E-12	1	2.574E-12	0.0887	0.7709	
B-DOPE%	6.630E-10	1	6.630E-10	22.86	0.0004	
C-FRR	8.013E-12	1	8.013E-12	0.2763	0.6087	
Residual	3.480E-10	12	2.900E-11			
Lack of Fit	2.014E-10	9	2.238E-11	0.4578	0.8404	not significant
Pure Error	1.466E-10	3	4.888E-11			
Cor Total	1.022E-09	15				

Supplementary Table S4. ANOVA for EE%.

Source	*Sum of Squares*	*df*	*Mean Square*	*F-value*	*p-value*	
**Model**	0.0853	5	0.0171	81.56	< 0.0001	significant
A-DOPA%	0.0526	1	0.0526	251.70	< 0.0001	
B-DOPE%	0.0165	1	0.0165	78.95	< 0.0001	
C-FRR	0.0032	1	0.0032	15.25	0.0029	
AC	0.0058	1	0.0058	27.93	0.0004	
BC	0.0071	1	0.0071	33.96	0.0002	
Residual	0.0021	10	0.0002			
Lack of Fit	0.0015	7	0.0002	1.17	0.4935	not significant
Pure Error	0.0006	3	0.0002			
Cor Total	0.0874	15				

Supplementary Table S5. ANOVA for D/L ratio.

Source	*Sum of squares*	*df*	*Mean square*	*F-value*	*p-value*	
Model	0.0223	2	0.0111	105.86	< 0.0001	significant
A-DOPA%	0.0058	1	0.0058	55.11	< 0.0001	
C-FRR	0.0165	1	0.0165	156.62	< 0.0001	
Residual	0.0014	13	0.0001			
Lack of Fit	0.0009	10	0.0001	0.6264	0.7486	not significant
Pure Error	0.0004	3	0.0001			
Cor Total	0.0236	15				

Supplementary Table S6. ANOVA for stability.

Source	*Sum of squares*	*df*	*Mean square*	*F-value*	*p-value*	
Model	0.0113	5	0.0023	45.80	< 0.0001	significant
A-DOPA%	0.0003	1	0.0003	5.43	0.0420	
B-DOPE%	0.0038	1	0.0038	76.99	< 0.0001	
C-FRR	0.0000	1	0.0000	0.3178	0.5853	
AC	0.0044	1	0.0044	89.37	< 0.0001	
B^2	0.0028	1	0.0028	56.89	< 0.0001	
Residual	0.0005	10	0.0000			
Lack of Fit	0.0004	7	0.0001	2.38	0.2556	not significant
Pure Error	0.0001	3	0.0000			
Cor Total	0.0118	15				

Supplementary Table S7. ANOVA for normalized drug release.

Source	*Sum of squares*	*df*	*Mean square*	*F-value*	*p-value*	
Model	4.55	5	0.9109	63.54	< 0.0001	significant
A-DOPA%	2.47	1	2.47	172.37	< 0.0001	
B-DOPE%	1.16	1	1.16	80.58	< 0.0001	
C-FRR	0.0483	1	0.0483	3.37	0.0962	
AB	0.4336	1	0.4336	30.24	0.0003	
AC	0.4461	1	0.4461	31.12	0.0002	
Residual	0.1434	10	0.0143			
Lack of Fit	0.1186	7	0.0169	2.05	0.2980	not significant
Pure Error	0.0248	3	0.0083			
Cor Total	4.70	15				

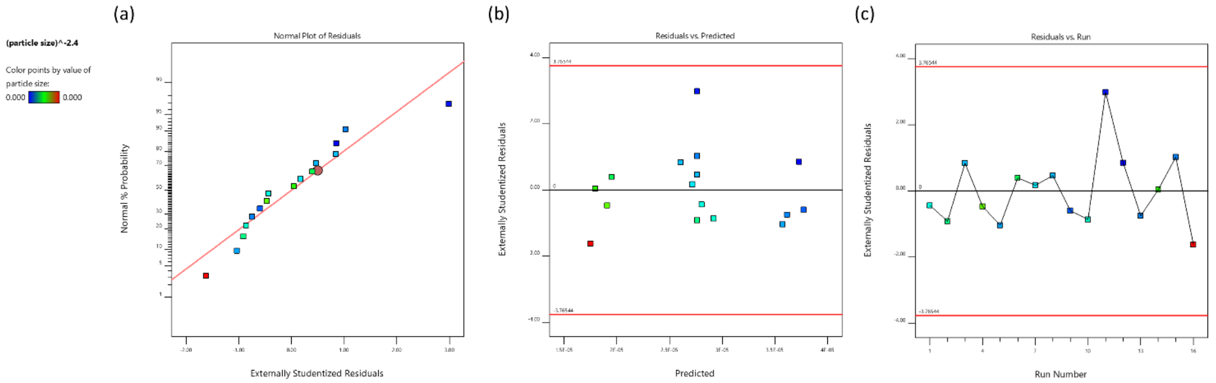

Supplementary Figure S1. The diagnostic plots for the validation of obtained model for particle size: (a) normal plot of residuals, (b) residuals versus predicted and (c) residuals versus run order.

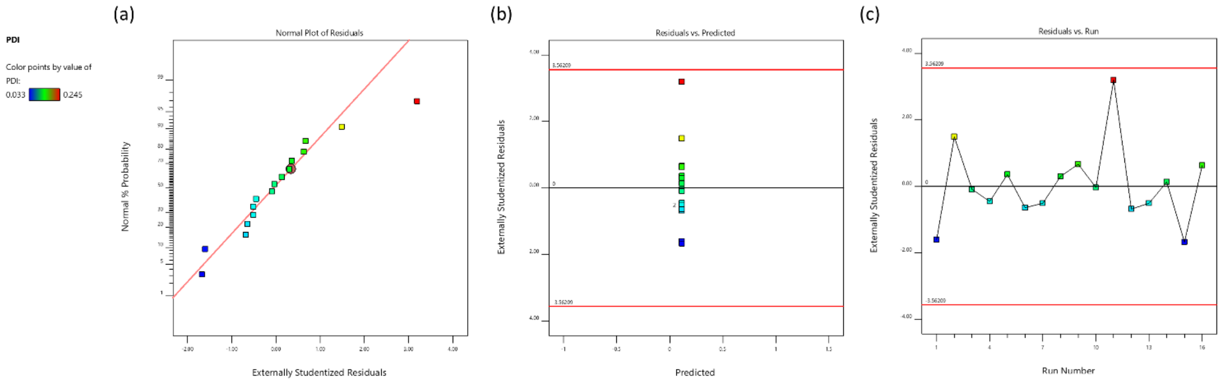

Supplementary Figure S2. The diagnostic plots for the validation of obtained model for PDI: (a) normal plot of residuals, (b) residuals versus predicted and (c) residuals versus run order.

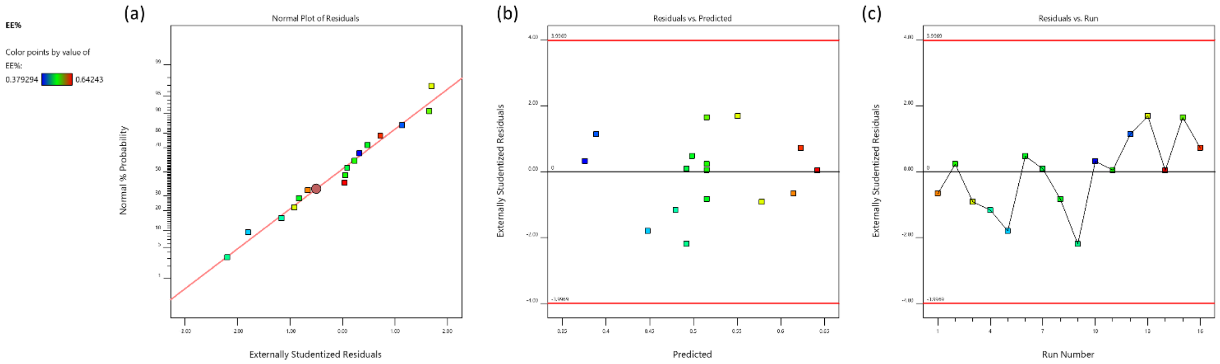

Supplementary Figure S3. The diagnostic plots for validation of obtained model for EE%: (a) normal plot of residuals, (b) residuals versus predicted and (c) residuals versus run order.

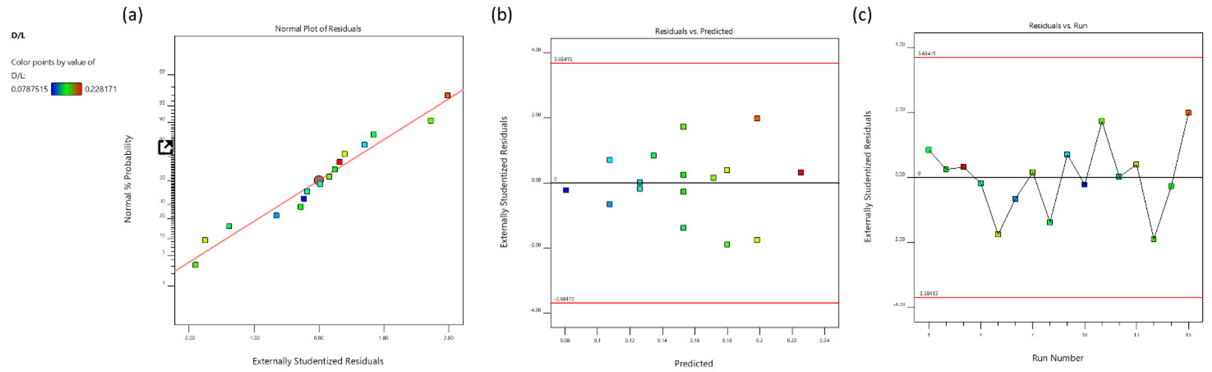

Supplementary Figure S4. The diagnostic plots for the validation of obtained model for D/L ratio: (a) normal plot of residuals, (b) residuals versus predicted and (c) residuals versus run order.

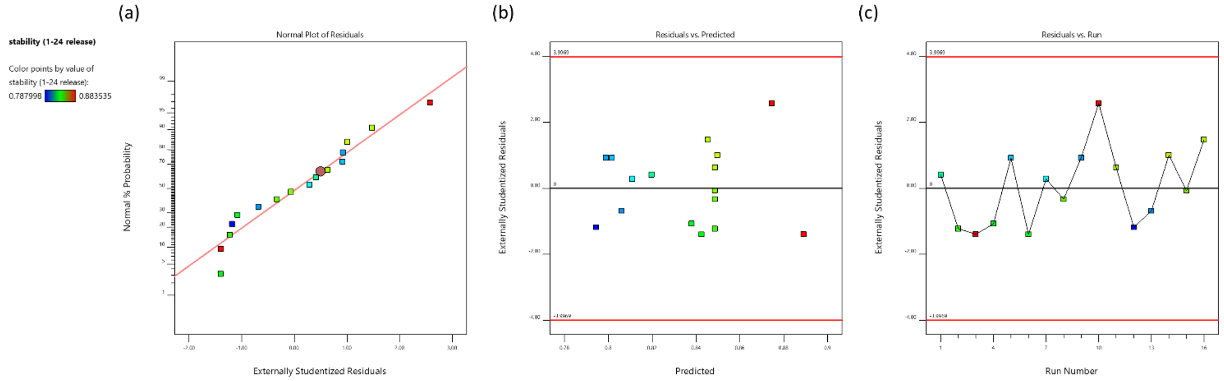

Supplementary Figure S5. The diagnostic plots for the validation of obtained model for stability: (a) normal plot of residuals, (b) residuals versus predicted and (c) residuals versus run order.

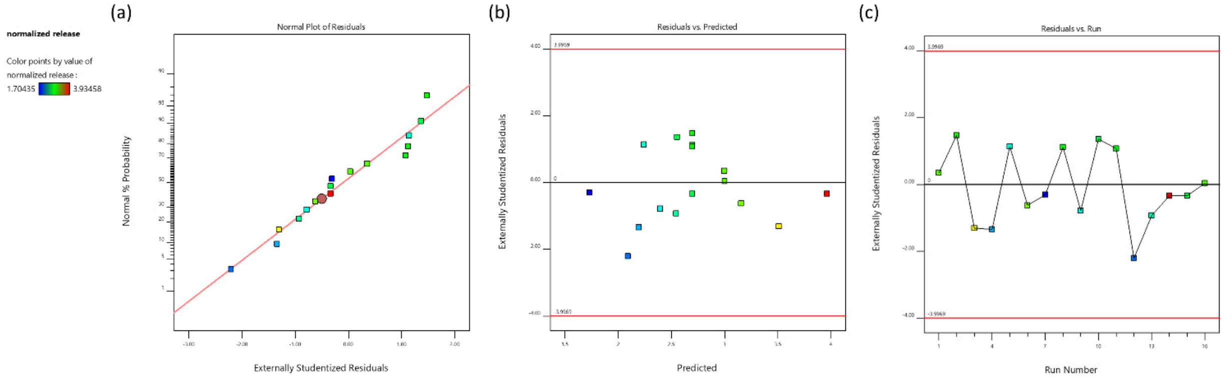

Supplementary Figure S6. The diagnostic plots for the validation of obtained model for normalized drug release: (a) normal plot of residuals, (b) residuals versus predicted and (c) residuals versus run order.
